# Analyzing the dose-dependence of the *Saccharomyces cerevisiae *global transcriptional response to methyl methanesulfonate and ionizing radiation

**DOI:** 10.1186/1471-2164-7-305

**Published:** 2006-12-01

**Authors:** Michael G Benton, Swetha Somasundaram, Jeremy D Glasner, Sean P Palecek

**Affiliations:** 1 Department of Chemical & Biological Engineering, 1415 Engineering Drive, University of Wisconsin, Madison, WI 53706, USA; 2 Genome Center of Wisconsin, 425 Henry Mall, University of Wisconsin, Madison, WI 53706, USA

## Abstract

**Background:**

One of the most crucial tasks for a cell to ensure its long term survival is preserving the integrity of its genetic heritage via maintenance of DNA structure and sequence. While the DNA damage response in the yeast *Saccharomyces cerevisiae*, a model eukaryotic organism, has been extensively studied, much remains to be elucidated about how the organism senses and responds to different types and doses of DNA damage. We have measured the global transcriptional response of *S. cerevisiae *to multiple doses of two representative DNA damaging agents, methyl methanesulfonate (MMS) and gamma radiation.

**Results:**

Hierarchical clustering of genes with a statistically significant change in transcription illustrated the differences in the cellular responses to MMS and gamma radiation. Overall, MMS produced a larger transcriptional response than gamma radiation, and many of the genes modulated in response to MMS are involved in protein and translational regulation. Several clusters of coregulated genes whose responses varied with DNA damaging agent dose were identified. Perhaps the most interesting cluster contained four genes exhibiting biphasic induction in response to MMS dose. All of the genes (*DUN1*, *RNR2*, *RNR4*, and *HUG1*) are involved in the Mec1p kinase pathway known to respond to MMS, presumably due to stalled DNA replication forks. The biphasic responses of these genes suggest that the pathway is induced at lower levels as MMS dose increases. The genes in this cluster with a threefold or greater transcriptional response to gamma radiation all showed an increased induction with increasing gamma radiation dosage.

**Conclusion:**

Analyzing genome-wide transcriptional changes to multiple doses of external stresses enabled the identification of cellular responses that are modulated by magnitude of the stress, providing insights into how a cell deals with genotoxicity.

## Background

DNA is the conduit for the transmission of genetic information between generations of a species [1]. The linear sequence of DNA is labile and can be altered by both intracellular and extracellular sources in a variety of ways. For example, within a cell errors by replication machinery can lead to point mutations while the physical stresses of replication can fracture strands of DNA. External sources can mutate DNA by alkylating single strands, causing interstrand crosslinks, or even breaking strands apart, resulting in the loss of some bases upon ligation [2-4].

One of a cell's most crucial tasks is maintaining the integrity of genetic information from one generation to the next [5]. Although some mutations are beneficial in the long term by allowing for adaptation to changing environments via natural selection, most mutations are detrimental and can lead to consequences as serious as cell death or transformation; the correlation between mutagenic activity and carcinogenic activity is approximately 90% [6]. This high correlation has increased interest in identifying causes of DNA damage and the effects of this damage on the cellular environment with the hopes of both preventing cancer and developing better chemotherapeutic agents for the treatment of existing cancerous cells [7-10].

*Saccharomyces cerevisiae *is commonly used as a model organism for studying the DNA damage response because its genome is well-annotated and the cells are easily cultured and genetically manipulated. Furthermore, because yeasts are eukaryotic, the gene regulation and biochemical pathways involved in responding to DNA damage are expected to be similar to, although perhaps simpler than, those of higher organisms. Indeed, the basic cellular responses to DNA damage have been shown to be similar in yeast and humans [11,12]. Many DNA damage sensory and repair mechanisms in humans have been identified from their homology to similar mechanisms in yeast [13].

The study of the DNA damage response in yeast has uncovered a broad variety of mechanisms used by the cells to sense and repair the damage. When cells sense a defect in their DNA, several signal transduction pathways are induced which in turn activate various checkpoints that cause cell-cycle arrest at the G1, S, or G2 phases until the defect can be repaired [14]. These signal transduction pathways regulate genome-wide expression patterns, causing hundreds of genes to be induced or repressed in direct response to the perceived damage [15]. Many of the induced genes are also upregulated in response to cell cycle arrest in stationary phase or in response to general cell stresses, indicating that DNA damage response pathways in yeast overlap with other cell cycle pathways [16,17]. Once cell division is arrested, repairs can be made in a variety of ways depending on the type of damage which has occurred. Single-strand lesions can be repaired by base excision repair, nucleotide excision repair, or mismatch repair using the non-altered strand as a template, while double-strand breaks are repaired by homologous recombination or non-homologous end joining [18-22].

To better define the cellular pathways involved in the recognition and repair of DNA damage, it is important to identify the global set of genes induced or repressed in response to the damage. These genes have been identified in a number of ways. Traditionally, pools of mutants were screened for sensitivity to various DNA damaging agents, enabling the proposal of epistasis groups based on common responses [23,24]. In the last 10 years, the proliferation of spotted and oligonucleotide microarrays have enabled genome-wide profiling in response to multiple DNA damaging agents [15,25-27]. These types of studies identified hundreds of genes not previously known to be modulated in response to DNA damage. Recently the two approaches have been combined by culture of gene deletion mutant strains tagged with unique identification sequences in the presence of a variety of DNA damaging agents, followed by PCR amplification and microarray hybridization of the identification sequences to assess the relative fitness level of each strain as a result of the exposure [28-31]. With each new study, genes not previously known to be regulated by DNA damage are identified, suggesting that there are additional genes and pathways whose control is crucial in DNA damage response yet to be distinguished.

In this study, we identified genes involved in the transcriptional response to DNA damage based on variation in expression upon exposure to two representative DNA damaging agents, methylmethane sulfonate (MMS) and ionizing gamma radiation (γ-ray), over three orders of magnitude of agent dose. MMS is a DNA alkylating agent known to methylate guanine residues at the N-7 position [30,32]. While this methylation is often tolerable to the cells, MMS can also methylate guanine at the O-6 position or adenine at the N-3 site [30,32]. These latter lesions inhibit DNA synthesis and must be repaired for replication to properly occur [30,32]. Ionizing radiation induces multiple types of DNA damage, but double strand breaks are considered to be the most lethal [33]. MMS generally produces single-strand lesions which inhibit DNA replication at the S phase checkpoint, while double-strand breaks caused by γ-ray inhibit the G_2_/M checkpoint [34]. Using spotted microarrays, Gasch et al. performed studies where *S. cerevisiae *cells were cultured in the presence of 0.02% MMS and the transcriptional response was assessed at multiple timepoints up to 120 min [26]. Jelinsky et al. performed a similar work using oligonucleotide arrays to monitor the transcriptional response of *S. cerevisiae *to 0.1% MMS at three timepoints up to 60 min [15]. Both groups identified clusters of genes co-regulated temporally in response to MMS.

Collectively, studies of the *S. cerevisiae *transcriptional response to MMS have reported results for multiple MMS doses. Jelinsky et al. reported that 693 genes showed a significant transcriptional modulation in response to 0.1% MMS for one hr [15], while Gasch et al. reported that more than 750 genes showed a significant change in transcription levels when exposed to 0.02% MMS for two hr [26]. More recently, using oligonucleotide arrays, Caba et al. identified 1,377 genes with a significant transcriptional response to 0.12% MMS for two hr [25]. These results suggest a dose-dependent element to the transcriptional response of *S. cerevisiae *to MMS, although yeast strains and culture conditions varied in these studies. Jelinsky et al. did examine multiple doses and reported a total of 1,863 genes responding to at least one of three doses; however, these doses varied by less than one order of magnitude [15].

Similarly, Jelinsky et al. observed 248 genes with a significant transcriptional response to 300 Gy of gamma irradiation [15]. Gasch et al. reported over 500 genes whose expression changed as a result of exposure to 170 Gy of gamma irradiation [26]. De Sanctis et al. used spotted microarrays to compare the global transcriptional responses of wild-type and *rad53 *strains to ionizing radiation administered in doses of 0.8 Gy, 8 Gy, 80 Gy, 300 Gy, and 400 Gy [35]. They observed 72 genes with a 2-fold or more change in transcription level in response to 80 Gy (with 56 genes being induced and 16 genes being repressed) and 86 genes with a 2-fold or more change in transcription level in response to 400 Gy. Recently, Mercier et al. used spotted microarrays to identify approximately 1400 genes with a significant change in transcription level after exposure to 200 Gy of ionizing radiation [34]. In this work, we used oligonucleotide microarrays to quantify the transcriptional response of *S. cerevisiae *cells to multiple doses, varying over three orders of magnitude, of MMS and γ-ray to show how the global response varies with dose and to identify sets of genes that respond similarly to dose changes.

## Results and Discussion

### Hierarchical clustering of array data

**Figure 1:**
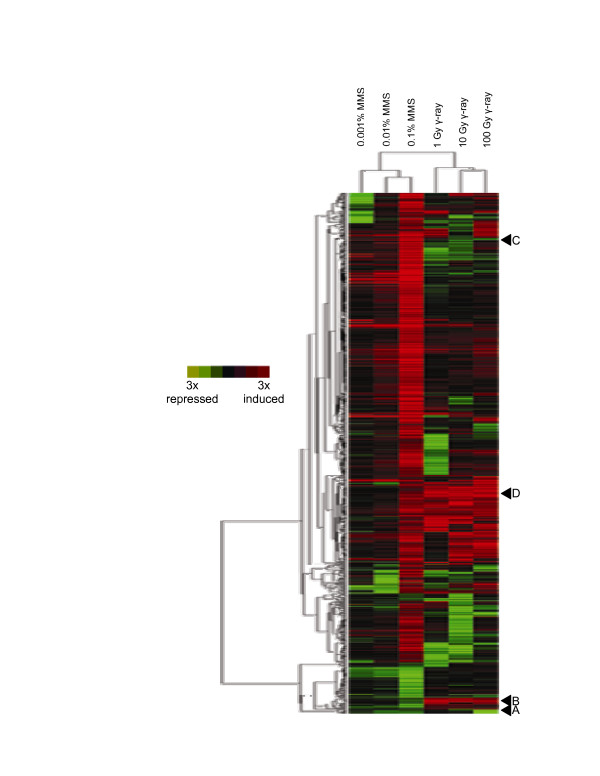
**Hierarchical clustering of 920 *Saccharomyces cerevisiae *genes with a significant transcriptional response to MMS and/or γ-ray**. *S. cerevisiae *cultures were exposed to one of three doses of MMS (0,001%, 0.01%, or 0.1% (v/v)) or one of three doses of γ-ray (1 Gy, 10 Gy, or 100 Gy) and their global transcriptional response was compared to that of control cells not exposed to DNA damaging agents. All genes clustered had at least a 3-fold change in expression level to one or more experimental conditions and a FDR value < 0.05 for at least one condition. Clustering of the experiments led to the most similar exposure conditions being grouped together. Four clusters are highlighted: (A) genes repressed by MMS and γ-ray, (B) genes repressed by MMS and induced by γ-ray, (C) genes induced by MMS and repressed by γ-ray, and (D) genes induced by both MMS and γ-ray.

**Figure 2:**
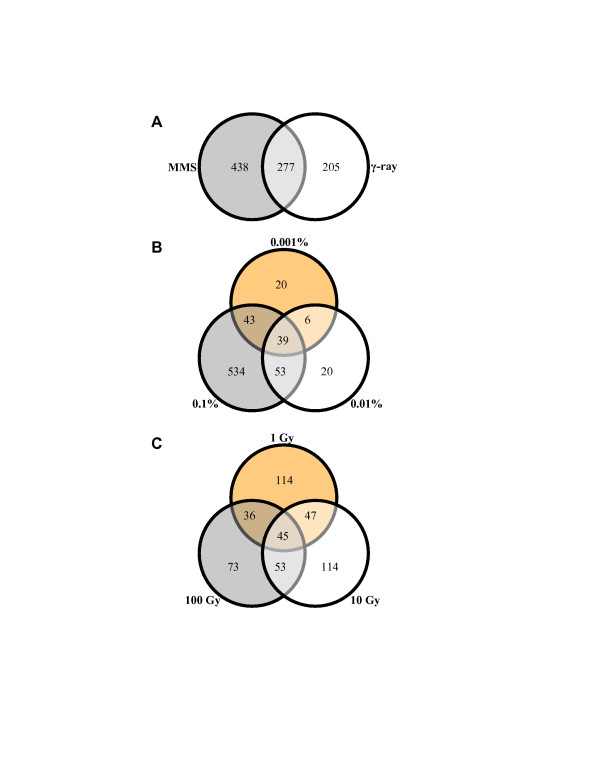
**Venn diagrams of genes responsive to MMS and/or γ-ray**. *S. cerevisiae *cultures were exposed to one of three doses of MMS (0,001%, 0.01%, or 0.1% (v/v)) or one of three doses of γ-ray (1 Gy, 10 Gy, or 100 Gy) and their global transcriptional response was compared to that of control cells not exposed to any DNA damaging agents. All genes in this diagram had at least a 3-fold change in expression level to one or more experimental conditions. (A) 920 genes responding to at least one dose of MMS and/or γ-ray, (B) 714 genes with at least a 3-fold response to at least one dose of MMS, (C) 482 genes with at least a 3-fold response to at least one dose of γ-ray.

**Figure 3:**
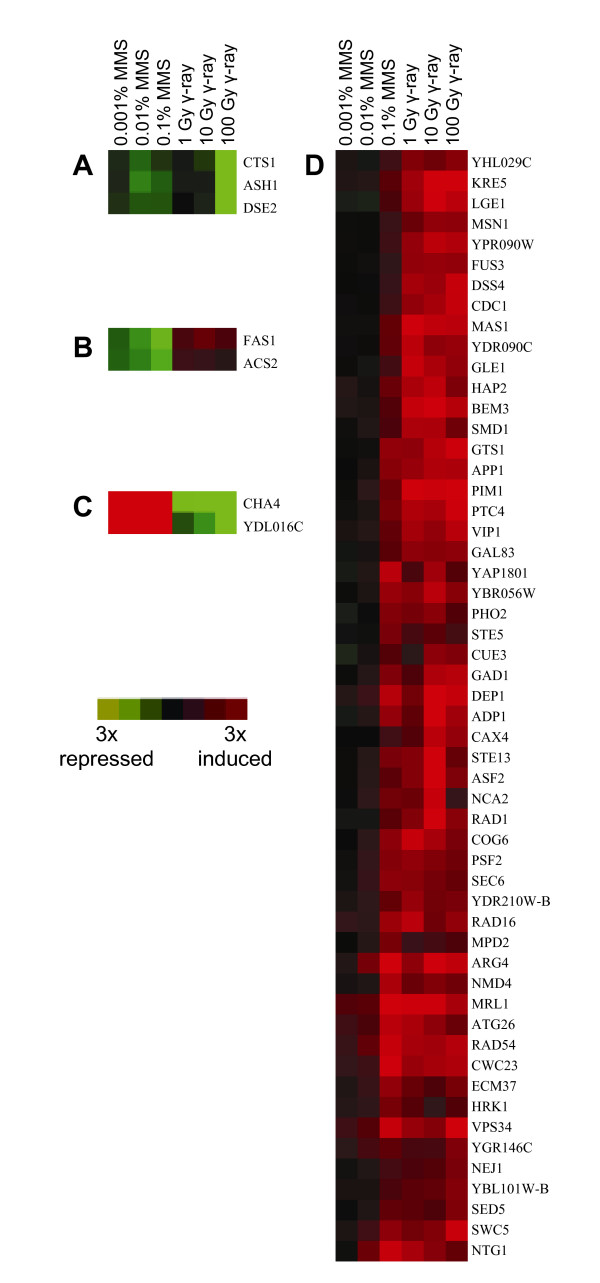
**Four clusters illustrating transcriptional responses to MMS and/or γ-ray**. *S. cerevisiae *cultures were exposed to one of three doses of MMS (0,001%, 0.01%, or 0.1% (v/v)) or one of three doses of γ-ray (1 Gy, 10 Gy, or 100 Gy) and their global transcriptional response was compared to that of control cells not exposed to any DNA damaging agents. All genes clustered had at least a 3-fold change in expression level to one or more experimental conditions. These four clusters had particularly interesting responses to MMS and γ-ray: (A) 3 genes repressed by all doses of MMS and γ-ray, (B) 2 genes repressed by all doses of MMS and induced by all doses of γ-ray, (C) 2 genes induced by all doses of MMS and repressed by γ-ray and (D) 54 genes induced by all doses of MMS and γ-ray.

**Figure 4:**
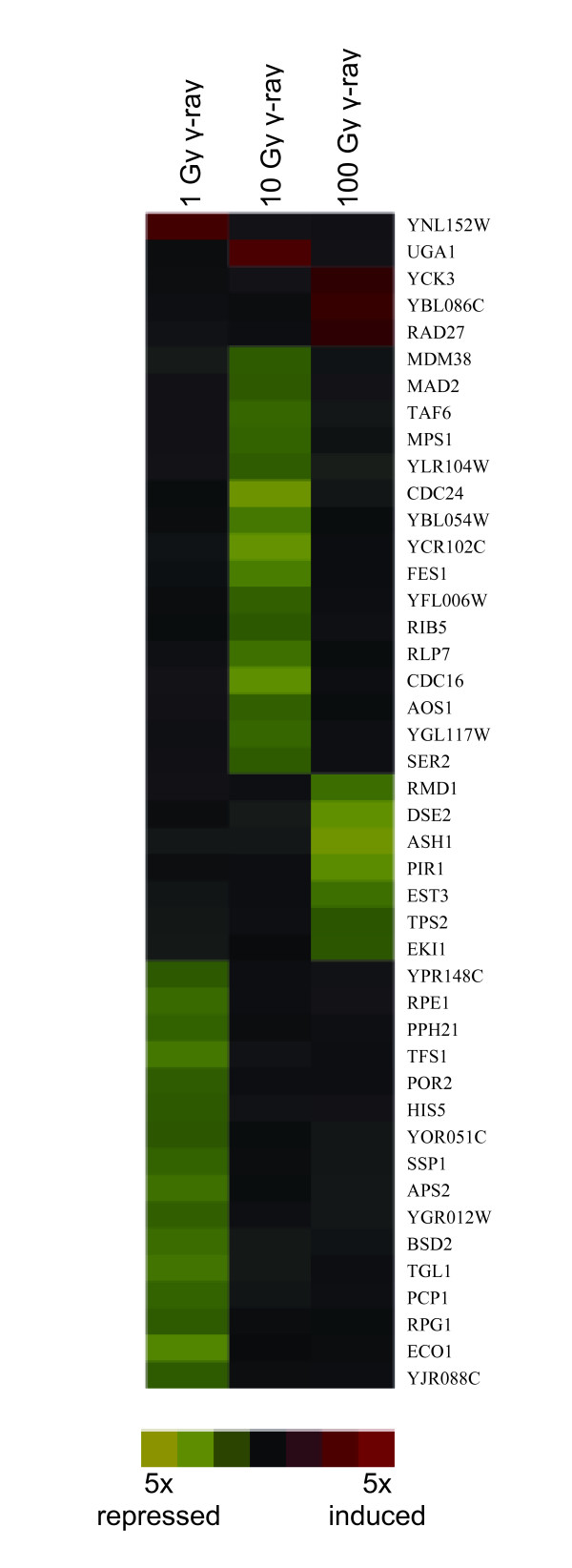
**Genes responding to only one dose of γ-ray**. Hierarchical clustering of the transcriptional response of S. cerevisiae after exposure to one of three doses of γ-ray (1 Gy, 10 Gy, or 100 Gy) identified 44 genes that showed at least a 3 fold transcriptional response to only one dose of γ-ray. Genes in this figure had at least a 3 fold modulation to one dose and less than a 1.4 fold modulation to all other doses.

**Figure 5:**
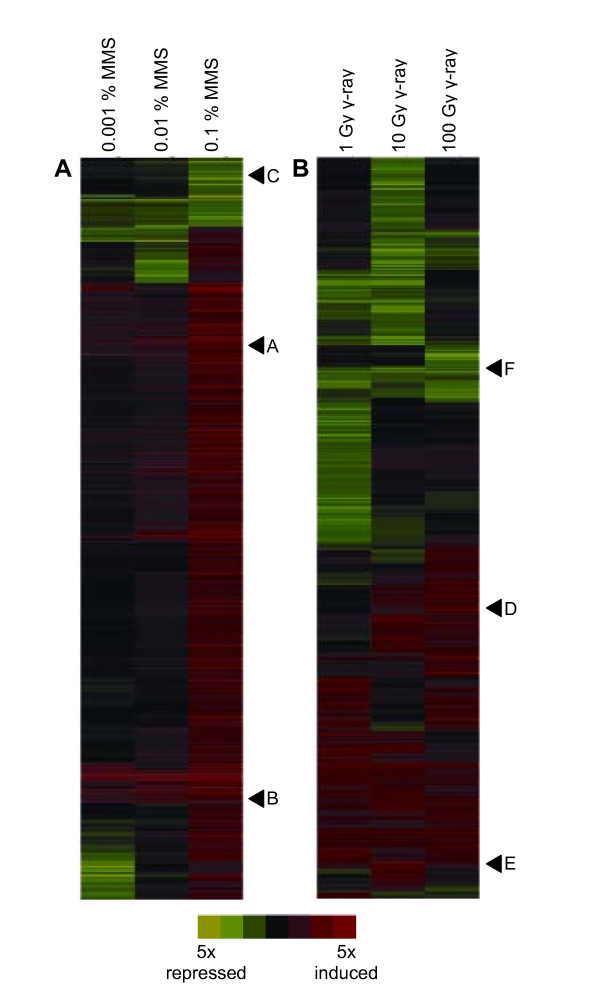
**Global transcriptional response to MMS or γ-ray**. (A) Hierarchical clustering of 714 genes with at least one transcriptional modulation of 3-fold or greater upon exposure to MMS. *S. cerevisiae *cultures were exposed to one of three doses of MMS (0,001%, 0.01%, or 0.1% (v/v)) and their global transcriptional response was compared to that of control cells not exposed to MMS. (B) Hierarchical clustering results for 482 *Saccharomyces cerevisiae *genes with at least one transcriptional modulation of 3-fold or greater in response to γ-ray exposure. *S. cerevisiae *cultures were exposed to one of three doses of γ-ray (1 Gy, 10 Gy, or 100 Gy) and their global transcriptional response was compared to that of control cells not exposed to γ-ray. Six smaller clusters (A-F) are highlighted because they showed a dose-dependent response (either monotonically increasing, monotonically decreasing, or biphasic) to either MMS or γ-ray.

**Figure 6:**
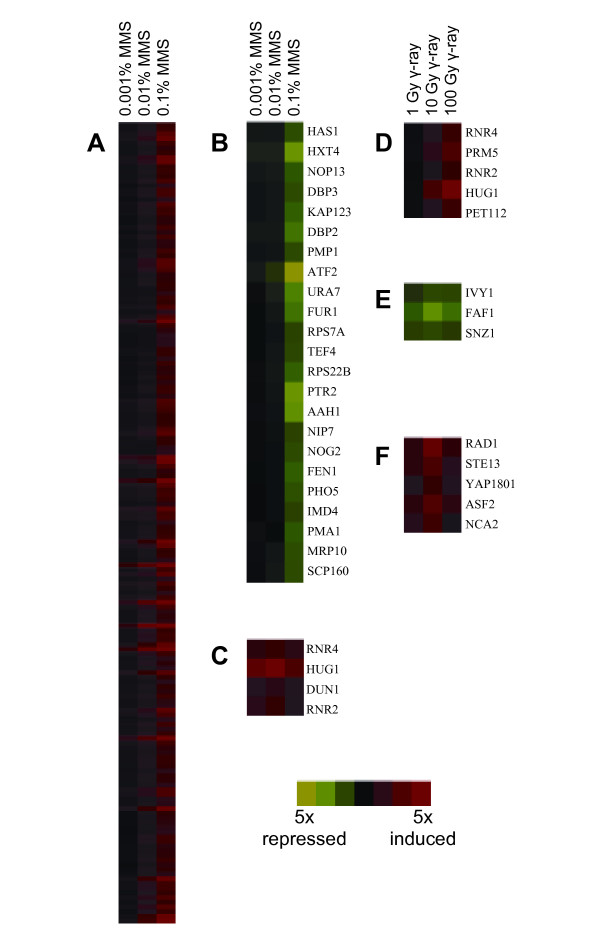
**Clusters with a dose-dependent response to MMS or γ-ray**. Six gene clusters with a dose dependent response (either monotonically increasing, monotonically decreasing, or biphasic) to either MMS or γ-ray. MMS doses tested were 0.001%, 0.01%, and 0.1%. γ-ray doses tested were 1 Gy, 10 Gy and 100 Gy. (A) monotonically increasing induction by MMS, (B) monotonically decreasing repression by MMS (C), biphasic induction by MMS, (D) monotonically increasing induction by γ-ray, (E) biphasic repression by γ-ray, and (F) biphasic induction by γ-ray.

Hierarchical clustering illustrating the transcriptional response of *S. cerevisiae *to MMS and γ-ray is shown in Figures 1, 2, 3, 4, 5, 6. Figure 1 shows all of the genes with a significant response to at least one dose of MMS and/or γ-ray, and Figure 2 shows a Venn diagram containing the number of genes regulated by each type and dose of genotoxicity. Figure 3 highlights four clusters found in Figure 1 that have notable responses to both MMS and γ-ray. Figure 4 illustrates a subset of genes that responded to only one dose of γ-ray. Figure 5-A contains only those genes with a significant transcriptional response to at least one dose of MMS (γ-ray was not considered in this cluster), and Figure 5-B contains genes with a significant response to at least one dose of γ-ray. Figure 6 contains clusters from Figure 5 in which a dose-dependent response to either MMS or γ-ray was observed.

### Global transcriptional response to MMS and γ-ray

Figure 1 shows an overview of the global transcriptional responses to both MMS and γ-ray, obtained by simultaneous clustering of genes and treatments. Clustering of treatments shows that all MMS experiments grouped together, as did the γ-ray-treated samples. Furthermore, within the cluster for a specific type of damage, the highest doses tested were clustered together (0.01% and 0.1% MMS and 10 Gy and 100 Gy for γ-ray). Clearly, exposure to MMS and γ-ray evokes a widespread transcriptional response in *S. cerevisiae *cells. Many of the clustered genes exhibited a transcriptional response to both MMS and γ-ray, although, it is clear that the response often varied with dose, as expected (see Additional Files 1, 2, 3, 4).

For further clarity, a Venn diagram (Figure 2) is presented to illustrate the number of genes modulated by at least  3-fold to each DNA damaging agent (2-A), each dose of MMS (2-B), and each dose of γ-ray (2-C).

### Clusters responsive to MMS and γ-ray

In addition to merely identifying genes that were modulated in response to MMS and/or γ-ray, we sought to identify clusters of genes that exhibited similar or opposite dose dependent responses to both MMS and γ-ray. Clustering diagrams of four clusters (A-D) highlighted in Figure 1 are shown in Figure 3.

Cluster 3-A contains 3 genes, all of which have been shown to play a role in mother-daughter cell separation and all of which were down-regulated in response to all doses of MMS and γ-ray. One of these genes is *CTS1*, a chitinase required for cell separation after mitosis, whose transcription is induced during the late M and early G_1 _stages of the cell cycle [36,37]. Microarray analysis of *S. cerevisiae *exposed to 5-fluorocytosine, an inhibitor of DNA synthesis, also identified down-regulation of *CTS1 *[38]. *DSE2 *is involved in the separation of daughter cells from mother cells by degrading the cell wall from the daughter side [39,40]. *ASH1 *localizes in daughter cells during mitosis and inhibits the transcription of HO, preventing the daughter cells from changing mating types [41]. The fact that the transcription factor *ASH1 *was downregulated after DNA damage exposure correlates well with observations of Workman et al. that Ash1p binds more genes in the absence of MMS than when MMS is present [42]. The co-regulation of these 3 genes implies that the cell may be actively trying to stop transmission of damaged DNA to daughter cells by inhibiting cell division.

Cluster 3-B contains 2 genes repressed in response to MMS and induced by γ-ray exposure. *FAS1 *is involved in the synthesis of fatty acids [43,44]. A third gene with similar behavior but excluded from this cluster due to higher than acceptable FDR values, *HMG1*, catalyzes the rate-limiting step in sterol biosynthesis [45]. Gasch et al. observed the repression of genes responsible for ergosterol synthesis in response to MMS [26], while De Sanctis et al. noted many of these same genes were induced in response to low doses of ionizing radiation, approximately 80 Gy or less [35].

Cluster 3-C contains 2 genes induced in response to MMS and repressed in response to γ-ray. One of the genes in this cluster, *YDL016C*, is a hypothetical ORF. The other gene, *CHA4*, is a transcription factor for *CHA1*. In the presence of serine or threonine as the sole nitrogen source, *CHA1 *is actively transcribed, but its expression is not detectable when cells are grown in the presence of other nitrogen sources such as ammonium [46]. A recent study by Yang et al. suggests *CHA4 *may play a role in cell-cycle regulation [47] as one of four putative interaction partners of the two forkhead transcription factors, *FKH1 *and *FKH2*, which are involved in cell-cycle regulation and mitotic exit and seem to play a role in yeast dimorphism [48]. This is particularly interesting since 5 of the genes clustered in Figure 1 (*ASH1*, *BEM3*, *CDC24*, *TEC1*, and *UBA4*) play a role in regulating pseudohyphal growth in *S. cerevisiae*.

The cluster highlighted in Figure 3-D is composed of 54 genes whose transcription is induced upon exposure to all doses of MMS and γ-ray used in this study. Several of the genes in this cluster are known to be involved in cellular responses to stress or DNA damage. *GAD1 *has been shown to mediate resistance to oxidative stress induced by hydrogen peroxide and diamide [49], and *NTG1 *has also been identified as crucial in repairing oxidative stress in DNA [50]. *RAD16 *is part of the nuclear excision repair pathway and has been shown to be involved in double-strand break repair in the fission yeast, *Schizosaccharomyces pombe*, suggesting an explanation for its induction in response to γ-ray [51]. *NEJ1 *is involved in the regulation of non-homologous end joining repair in response to double-strand breaks [52]. A potential reason for *NEJ1 *induction by MMS may involve creation of single-strand breaks following excision repair of methylated bases. When the replication fork confronts these breaks, double-strand breaks can be caused by the forces of replication [53], which could elicit a partial transcriptional response in genes responsible for repairing double-strand breaks. This could also address induction of *RAD54*, known to be crucial in homologous recombination for the repair of double-strand breaks [54], in response to MMS. The genes shown in this cluster suggest there is some overlap of DNA-damage repair pathways in sensing and responding to damage induced by MMS and γ-ray, as members of the base excision repair pathway, nucleotide excision repair pathway, non-homologous end joining pathway, and homologous recombination pathway were all induced.

To gain additional insight into the coregulation of Cluster 3-D, the MEME system was used to identify highly conserved motifs within the regions 500 base pairs upstream from all of the genes in the cluster [55]. Thirty seven of the genes contained a 16 bp region with an E-value of 0.05 (see Additional file 5). To our knowledge, this sequence (T-T- [CT]- [CA]- [CT]-C- [CT]- [CA]-T- [TC]- [TC]-T- [TC]-C- [TC]- [TA]) has not been shown to be a transcription factor binding site in *S. cerevisiae *[56,57]. Although these 37 genes are involved in a wide variety of biological processes, it is possible that this conserved motif comprises or contains a site to which a transcription factor induced by DNA-damage can bind and simultaneously regulate several genes induced by both MMS and γ-ray, but clearly further experiments are required to prove this notion. A comparison of this putative transcription factor binding site with the JASPAR database showed closest homology with a mouse pancreatic development transcriptional repressor (81.78%) [58] and a human nuclear receptor ligand activated transcription factor (67.67%) [59].

### Genes modulated by only one dose of γ-ray

During analysis of the dose dependent transcriptional response to MMS and γ-ray, we sought to identify genes whose transcription was only modulated in response to a single dose of damaging agent. While all of the genes found to respond to only one dose of MMS responded only to the highest dose, 44 genes responded to only one of the γ-ray doses tested and interestingly some responded only to the lowest or to the intermediate dose (Figure 4).

Expression of 5 genes was induced by a single γ-ray dose, with perhaps the most interesting being *UGA1*. *UGA1 *is a γ-aminobutyrate transaminase that along with *GAD1*, discussed earlier, helps regulate the tolerance of cells to oxidative stresses [49]. Its induction at the low dose of 10 Gy indicates the sensitivity of this particular stress response in *S. cerevisiae*. Many more genes, 39, were repressed at only one dose of γ-ray. At the lowest dose tested, 16 genes were repressed, including 3 involved in protein synthesis regulation (*TFS1*, *BSD2*, and *PPH21*). This down-regulation is consistent with features of the environmental stress response in yeast [17]. Another interesting gene in this cluster is *ECO1*, which is required for cohesion of sister chromatids during DNA replication, a necessity for repair of double-strand breaks caused by replication [60]. The fact that 44 genes were induced or repressed at only one dose of MMS or γ-ray illustrates one benefit of analyzing genome-wide transcriptional changes to external stresses at multiple doses.

The MEME system was used to identify conserved motifs within the clusters shown in Figure 4. All 16 of the genes induced by 1 Gy of γ-ray conserved a similar 12 bp sequence ([CG]-T-T-T-T-T-T-T- [TC]- [TC]- [TA]-T) with an E-value of 0.08. This motif contains a reported binding site of Azf1p, TTTTTCTT [56,57]. Azf1p is a transcription factor shown to regulate cell cycle genes in response to DNA damage caused by MMS [42]. While Azf1p is not known to regulate all of the genes in this cluster, it is possible that Azf1p is involved in regulating these genes in response to DNA damage caused by γ-ray, but further experimentation is necessary to test this relationship. A comparison of this putative transcription factor binding site with the JASPAR database showed closest homology with a human (79.17%) [61] and a rat (80.45%) forkhead transcription factor [62].

### Global transcriptional response to either MMS or γ-ray

To identify genes that were coregulated at all doses of either MMS or γ-ray, the genes were reclustered such that MMS and γ-ray exposure were considered separately (Figure 5) (see Additional Files 3, 4, and 6). All of the genes in each cluster in Figure 5 had at least one transcriptional fold change of 3 or more. One of the most visually evident differences between Figure 5-A and 5-B is the relative number of genes induced or repressed by MMS and γ-ray. Of the 714 genes modulated by MMS (5-A), 581 (81%) are induced (when the dose yielding the highest modulation is considered). Of the 482 genes induced or repressed by γ-ray, 228 (47%) are induced. This suggests that cells modulate entire cellular processes differently based on the type of damage occurring. For example, MMS causes an increased repression of cellular transport and metabolism genes, while γ-ray causes an increased repression of protein regulation and translation genes (see Additional file 6).

Comparing genes with roles in DNA replication/repair and cell-cycle progression and how they were differentially modulated by MMS and γ-ray provides insight into cell responses to these agents. In the cluster shown in Figure 1, a total of 85 genes fell into the DNA replication/repair and cell-cycle progression category, 4 of which are nucleotide excision repair pathway genes. In the MMS cluster (Figure 5-A), a total of 63 genes fell into this category, and all 4 nucleotide excision repair pathway genes are present. In the γ-ray cluster (Figure 5-B), 59 genes fell into the DNA replication/repair and cell-cycle progression category, but only one of the nucleotide excision repair genes, *RAD16*, was significantly modulated. This variation suggests that, as would be expected, the nucleotide excision repair pathway is much more active in response to MMS than γ-ray.

An interesting subset of the genes involved in cellular metabolic processes includes 8 genes involved in ergosterol synthesis – *ERG28*, *ERG2*, *ERG11*, *ERG6*, *ERG25*, *ERG13*, *ERG1*, and *MVD1*. These genes all appear in the MMS and γ-ray cluster (Figure 1) and all 8 also appear in the MMS cluster (Figure 5-A), but none are present in the γ-ray cluster (Figure 5-B), suggesting a link between the MMS response and ergosterol synthesis. Gasch et al. previously noted four of these eight genes (along with several other ergosterol synthesis genes) were down-regulated in response to 0.02% MMS [26]. They attributed this regulation to the fact that many of these genes are regulated by Hap1p and suggested that Hap1p could be methylated by MMS. Jelinsky and Samson reported the repression of *ERG3*, a sterol desaturase, and *SUR4*, a sterol isomerase, upon exposure to 0.01% MMS [27].

Considering the role of *DAP1 *offers additional insight into the link between ergosterol synthesis and the MMS response. Hand et al. found that *dap1 *strains were extremely sensitive to MMS, but still underwent cell cycle arrest similar to wild type cells [63]. They proposed that *DAP1 *is involved in the response to MMS, but not in triggering cell-cycle arrest. *DAP1 *is also required for normal ergosterol levels in *S. cerevisiae*; however *dap1 *strains did not possess increased membrane permeability despite lower levels of ergosterol [63]. This demonstrated that the MMS sensitivity of *dap1 *was not due to a compromised membrane allowing more MMS inside the cell, but rather some other pathway interactions within the cell. The down-regulation of ergosterol synthesis genes in response to MMS may allow cells to divert energy from normal ergosterol synthesis levels to fighting the effects of MMS. An alternative explanation could be that the lack of cell division due to cell-cycle arrest has lessened the need for ergosterol. Neither of these explanations, however, explains why these genes are down regulated in response to MMS but not γ-ray.

### Clusters with dose-dependent responses to MMS or γ-ray

To illustrate the dose-dependent response of certain genes to either MMS or γ-ray, six smaller clusters from Figure 5 in which one of three specific dose-dependent responses was observed: monotonically increasing, monotonically decreasing, or biphasic (where peak induction or repression occurred at the intermediate dose) were identified and are presented in Figure 6.

Cluster 6-A contains 171 genes which exhibit monotonically increasing expression in response to MMS: the genes are induced at all doses of MMS tested and the level of induction increases with MMS dose. Four of the genes in this cluster are directly involved in sensing or repairing DNA damage – *MAG1*, *HSM3*, *YIM1*, and *DDR48*. *HSM3 *is involved in DNA mismatch repair in slowly dividing cells [64,65], *YIM1 *is believed to be involved in the cellular response to DNA-damaging agents [66], and *DDR48 *is induced in response to heat shock or DNA-lesion producing treatments [67]. Additionally, ten other genes *(LCB5, GLR1, GPX2, YAP1, GRE2, SSA4*, *PRE1*, *PRE3*, *UBI4*, and *YNR064C*) involved in stress response were also monotonically induced by escalating MMS concentrations. *YAP1 *is particularly intriguing because it is considered to be the primary regulator of the oxidative stress response [68], and it serves as a transcription factor for multiple genes and other transcription factors during the MMS response [42]. *PRE1 *has recently been shown to be modulated in response to MMS by the transcription factor Swi6p [42]. Another interesting feature of cluster A is that 26 of the genes are involved in protein ubiquitination and/or catabolism. MMS induction of protein degradation has been observed in yeast and higher organisms [27,69] and may be related to the degradation of methylated proteins.

Cluster 6-B contains 23 genes which exhibit monotonically decreasing repression with increasing MMS concentration. The largest subset within this cluster is a group of 6 genes (*PTR2*, *RPS7A*, *RPS22B*, *MRP10*, *KAP123*, and *NOG2*) involved in protein synthesis or transport. *PTR2 *has been shown to be down-regulated as part of the stress response initiated by MMS exposure [42]. This correlates well with the observation stated earlier: in response to environmental stress, many genes involved in protein synthesis and metabolism in *S. cerevisiae *are repressed [26].

The MEME system was used to identify conserved motifs within cluster 6-B. Thirteen of the genes in this cluster conserved a 14 bp region (T-G-C-G-A-T-G-A-G- [CA]- [TA]- [GT]-A-G) with an E-value of 1.2 E-10. Within this motif is a highly conserved 9 bp region proposed to be a binding site for Cha4p (TGCGATGAG) [57]. However, to date Cha4p has not been shown to regulate any of the genes in this cluster [70], and *CHA4 *expression is upregulated at all MMS doses tested in this study (Figure 3-C). A comparison of this putative transcription factor binding site with the JASPAR database showed closest homology with a mouse pancreatic development transcriptional repressor (72.57%)[58] and a human zinc finger transcription factor involved in the differentiation response to Ras in human carcinomas (67.67%) [71].

Cluster 6-C, a group of four genes with a biphasic induction in response to MMS treatment, is very intriguing. All of the genes in this cluster are involved in the Mec1p kinase pathway known to respond to DNA damage caused by MMS. Ribonucleotide reductase is an enzyme that consists of four subunits, two large (*RNR1 *and *RNR3*) and two small (*RNR2 *and *RNR4*), and catalyzes the rate-limiting step of dNTP synthesis [72,73]. An increase in dNTP levels is often associated with DNA damage and is considered necessary for DNA repair and resumption of normal transcription [74]. When a cell senses genotoxicity or a stalled replication fork, the signal is transduced through the kinase checkpoint Mec1p, leading to the activation of the kinases Rad53p and Dun1p [75]. Dun1p plays a role in the phosphorylation and eventual degradation of Sml1p, an inhibitor of RNR gene transcription [76]. When Sml1p is destroyed, RNR genes are transcribed and cellular dNTP levels rise, leading to a resumption of DNA synthesis. *HUG1*, also transcribed in a kinase checkpoint dependent manner, is downstream from *DUN1 *and thought to play a role in the recovery from the transcriptional response to DNA damage and cell-cycle arrest [77]. The biphasic induction of *DUN1, RNR2, RNR4*, and *HUG1 *was unexpected and may suggest that the Mec1p pathway is eventually scaled back or even shut off at high doses of MMS. *RNR1 *and *RNR3 *were also biphasically induced in response to MMS but were excluded from clustering analyses because of prohibitive FDR analysis results.

There are several possibilities for the decreased induction of genes involved in the Mec1p pathway at the highest MMS doses. First, there may be alternative cellular responses to different MMS doses. Gasch et al. reported that *mec1 *mutants show an induction of *RNR2*, *RNR4*, and *DUN1 *in response to DNA damage, although the response is muted, indicating there may be other mechanisms to derepress the RNR genes [26]. More recently, Dubacq and coworkers described a Snf1p kinase directed pathway activated in response to three DNA-damaging agents that cause stalled replication forks independently of and in parallel to the *MEC1 *pathway [78]. They described *snf1 *mutants that were sensitive to hydroxyurea, MMS (0.02% and 0.04%), and cadmium ions but not to UV radiation, γ-ray, hydrogen peroxide, camptothecin, and phleomycin. Based on these results, the activity of the Snf1p kinase mediated pathway in response to higher doses of MMS warrants further study.

A second possibility addressing the biphasic response is that MMS may methylate a member of the Mec1p pathway, leading to its inactivation or destruction. In our experiments, *RAD53 *showed a monotonically increasing induction in response to MMS treatments above 0.001%, although it was excluded from our clustering analysis as a result of FDR filtering. This fact in conjunction with the cluster shown in Figure 6-C suggests the possibility of a failure in the Mec1p pathway between Rad53 and Dun1. This could have been caused by methylation of a protein with susceptibility to MMS at high doses that was degraded in response to its alkylation by MMS effectively creating a roadblock in the DNA-damage response.

A final possibility, though perhaps less likely, it that the RNR enzyme itself is methylated by MMS, leading to its inability to catalyze dNTP synthesis. Although MMS has been shown to increase dNTP levels at low doses [74], it is possible that RNR is alkylated at higher doses since RNR has an active cysteine residue that is involved in the catalysis of dNTP synthesis [78]. Once a minimum MMS concentration threshold is passed, RNR could be rendered incapable of catalyzing dNTP synthesis and dNTP levels in the cell would drop. This drop in dNTP concentration could be sensed by the cell and mistakenly interpreted as a completion of DNA synthesis, thus inactivating the Mec1p kinase pathway.

Cluster 6-D contains 5 genes that are induced in a monotonically increasing manner in response to γ-ray. *PRM5 *has been shown to be induced in response to compromised cell wall integrity [79]. The responses of *RNR4*, *RNR2*, and *HUG1 *shown in this cluster are very interesting. All of these genes were induced at all doses of γ-ray, with the highest induction coming at the highest dose. *RNR1 *and *RNR3 *are also achieve maximum induction by γ-ray at the highest dose, but were excluded from this clustering analysis by statistical filtering. This behavior offers an interesting contrast to the biphasic response these genes exhibited upon MMS exposure.

The final two clusters shown in Figure 6 (6-E and 6-F) both show a biphasic response to γ-ray. The three genes in 6-E are repressed, while the five genes in cluster 6-F are biphasically induced by γ-ray. An interesting member of this cluster is *RAD1*, which functions in the repair of double strand breaks that lack overlapping end sequences [80].

**Figure 7:**
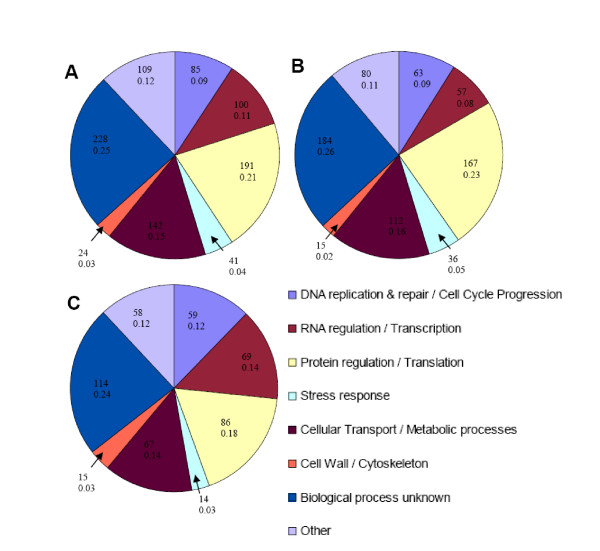
**GO annotations of responsive genes**. (A) Summary of the GO biological process for each of the 920 *S. cerevisiae *genes with a statistically significant response to MMS and/or γ-ray [81]. The majority of the genes with known function are involved in DNA synthesis or repair, transcription, or translation and indicate a massive cellular response to MMS and/or γ-ray. (B) Summary of the GO biological process for each of the 714 genes with a 3-fold transcriptional modulation in response to at least one MMS concentration tested. (C) Summary of the GO biological process annotations for each of the 482 genes with a 3-fold transcriptional modulation in response to at least one dose of γ-ray.

To analyze the types of genes modulated in response to the induced stresses, genes were divided into categories according to the GO annotation [81] of the biological processes in which they are known to be involved (Figure 7). Figure 7-A contains all 920 genes induced or repressed in response to MMS or γ-ray. Figure 7-B and 7-C contain the 714 genes responsive to at least one dose of MMS and 482 genes responsive to at least one dose of γ-ray, respectively. A comparison of the three panels illustrates how the transcriptional response of *S. cerevisiae *apparently varies with the source of DNA damage.

Approximately 25% of genes responding to either MMS or γ-ray were classified as biological process unknown (Figure 7-A). This number is reasonable considering 34% of *S. cerevisiae *predicted ORFs are currently unverified [82]. Within this category, 67% of the modulated genes were upregulated.

A second group of genes in Figure 7-A, approximately 9%, was composed of genes involved in DNA synthesis and repair or cell-cycle progression. Sixty-five percent of these genes were upregulated in response to DNA damage. Of the 85 genes classified as DNA synthesis and repair or cell-cycle progression genes, 24 are specifically involved in DNA damage repair, including *MAG1 *which was highly induced in response to MMS and is known to be a member of the base excision repair pathway involved in the repair of N7-alkylguanines, among the deleterious alkylations caused by MMS [83-87]. *RAD16 *and *RAD7*, which encode two parts of a heterotrimeric complex that removes damaged oligonucleotides during nucleotide excision repair [88], were also induced in response to MMS. *RNR2 *and *RNR4 *were highly induced in response to γ-ray; these genes have been shown to be transcriptionally induced in response to hydroxyurea and ionizing radiation [89]. A second subset of the DNA synthesis and repair or cell-cycle progression genes includes 9 genes (6 induced and 3 repressed) that function in cell-cycle checkpoints, such as *CDC53*, *HRT1*, and *PTK2*, all involved in the G_1_/S transition of the cell cycle. This regulation presumably halts cell cycle progression, preventing the synthesis of damaged DNA.

A third major category of genes whose expression changes upon MMS or γ-ray exposure includes those involved in RNA regulation and transcription. Of the 100 genes in this category, 57 were induced and 43 were repressed. Many of these genes are involved in mRNA catabolism, export from the nucleus, or polyadenylation. For example, *HRP1 *and *CLP1*, both induced in response to MMS, are subunits of cleavage factor I, required for the cleavage and polyadenylation of 3' mRNA ends [90]. Other RNA regulation and transcription genes modulated in response to MMS and γ-ray include those involved in the positive and negative RNA polymerase II promoter regulators, spliceosome activity, and ribosome synthesis. The modulation of these genes correlates well with the observation that the transcriptional response of genes involved in cell growth-related processes are regulated by the environmental stress response [17].

Another category of genes modulated by MMS and γ-ray includes genes involved in protein regulation and translation, accounting for 21% of the genes in Figure 7-A. Seventy-three percent of the genes in this category were induced. In response to environmental stress, expression many genes involved in protein synthesis and metabolism in *S. cerevisiae *changes, presumably to allow the cell to conserve energy [26].

Finally, a group of 41 genes involved in the response to various general cell stresses are modulated upon exposure to MMS and/or γ-ray. 38 of these genes were induced by more than 3 fold upon exposure to DNA damage. This group includes genes that respond to drugs, osmotic stress, various metal ions, and oxidative stress, indicating the regulation of many genes involved in general stress response pathways.

Figure 7-B, which shows the functional classification of genes with a 3 fold or greater transcriptional response to MMS only, shows little variation from panel A in the fraction of genes composing each functional category. Changes from panel A to panel C, which shows genes with at least a 3 fold transcriptional response to γ-ray only, were mildly more apparent. Additionally, the percentage of genes involved in DNA replication/repair and cell-cycle progression increased slightly while the fraction of genes involved in protein metabolism and translation and the fraction involved in general cellular metabolism decreased slightly. This change may be due to fewer cellular proteins being damaged by γ-ray than by MMS. This is supported by a comparison of panels B and C, which perhaps indicates that MMS elicits a broader response of genes involved in protein regulation and translation than does γ-ray, presumably from methylation and subsequent degradation of proteins following MMS exposure, leading to a higher rate of protein turnover in the cell.

## Conclusion

The global transcriptional response of *S. cerevisiae *as a function of dose of DNA-damaging agent was measured by exposing *S. cerevisiae *cells to multiple concentrations, varying over 3 orders of magnitude, of two representative DNA-damaging agents, MMS and γ-ray, and hybridizing their cRNA to oligonucleotide microarrays containing over 6,300 hypothetical and known *S. cerevisiae *ORFs. Hierarchical clustering of genes with a statistically significant change in transcription enabled the identification of several clusters of coregulated genes whose responses varied with DNA damaging agent dose. Our study showed many genes involved in sensing and repairing DNA damage are regulated by both MMS and γ-ray exposure and suggests interconnection between the pathways that respond to various types of DNA damage. There were differences in the responses as well. For example, 8 genes involved in the ergosterol synthesis pathway were down-regulated in response to MMS, but not significantly modulated by γ-ray exposure, verifying earlier observations [26,27]. Furthermore, we identified 44 genes which responded to only one dose of γ-ray, highlighting one benefit of measuring the transcriptional response to external stresses over multiple doses. MEME analysis identified conserved motifs upstream of multiple genes in these clusters, potentially signifying common regulatory elements.

Further analysis revealed 6 clusters of genes with a dose-dependent response to either MMS or γ-ray. Clusters were identified based on their mode of response, either monotonically increasing, monotonically decreasing, or biphasic (where peak modulation occurred at the intermediate MMS or γ-ray dose). Analyzing genome-wide transcriptional changes to multiple doses of external stresses enables the identification of cellular responses that are modulated by extent of damage, providing new insights into how a cell deals with genotoxicity. Perhaps the most interesting example of this was a cluster that contained 4 genes (*DUN1*, *RNR2*, *RNR4*, and *HUG1*) that are members of the Mec1p kinase pathway known to respond to MMS induced damage, presumably due to stalled DNA replication forks. These genes showed a biphasic induction in response to MMS treatment, while many of the same genes, *RNR2*, *RNR4*, and *HUG1*, were monotonically increasingly induced by γ-ray.

## Methods

### Strains and culture conditions

Three independent experiments were performed for each exposure condition. *S. cerevisiae *strain SPY810 (W303 *MAT a ura3-52 his3::hisG leu2::hisG*) was grown in rich medium (YPD) [91]. Cultures were inoculated overnight at 30°C and diluted 50 fold with YPD the next morning. Cells were returned to 30°C with shaking at approximately 250 rpm until mid log-phase growth was achieved.

### DNA damage induction

A culture of mid log-phase yeast cells (OD_600 _~ 0.5) was divided into four equal samples. Fresh MMS (Acros Organics, 99% pure, Fisher Scientific, Pittsburgh, PA, USA) was added to three of the samples to final concentrations of 0.001% (v/v), 0.01%, and 0.1%. These doses allowed for greater than 90% cell survival after one hour of exposure to all doses, as confirmed by flow cytometry quantifying fractions of cells excluding propidium iodide (see Additional file 7). The fourth sample had no MMS added but was otherwise handled identically to the MMS-treated cultures. After addition of MMS, cells were returned to 30°C incubation with light shaking for 60 min.

A second culture of mid log phase yeast cells was divided into four equal samples. Using a Cs^137 ^source (7 Gy/min), three of the aliquots were exposed at room temperature in YPD to ionizing radiation to final doses of 1 Gy, 10 Gy, and 100 Gy. These doses allowed for greater than 90% cell survival one hr following exposure, as confirmed by flow cytometry quantifying fractions of cells excluding propidium iodide (see Additional file 7). The fourth sample was not irradiated but was otherwise handled identically to the irradiated cultures. After irradiation, cells were returned to 30°C incubation with light shaking for 60 min.

### RNA isolation

For each of the culture conditions previously described, total RNA was recovered from approximately 5 × 10^7 ^*S. cerevisiae *cells using the RNeasy Mini Kit (Qiagen, Chatsworth, CA, USA). The manufacturer's protocol for enzymatic lysis was followed using 250 U of zymolyase (Associates of Cape Cod, Seikagaku America, Falmouth, MA, USA) per recovery column to generate spheroplasts. Once isolated from the spheroplasts, RNA integrity was examined using OD_260/280 _absorption ratio and agarose gel electrophoresis with ethidium bromide staining. RNA was stored in RNase free TE at -80°C.

### Microarray probe preparation

Microarray methods were modified from [92]. Total RNA was converted to double-stranded cDNA using the Superscript Double-Stranded cDNA synthesis kit from Invitrogen (Carlsbad, CA, USA) and a Proligo oligo dT primer (Sigma-Aldrich, St. Louis, MO, USA) containing a T7 RNA polymerase promoter region (5'-GGCCAGTGAATTGTAATACGACTCACTATAGGGAGGCGG-T_24_-3'). To synthesize the first strand of cDNA, 5 μg denatured total RNA was combined with 1× first strand buffer, 10 mM DTT, 500 μM (ea.) dNTPs, 5 pmol dT primer, and 600 U of SuperScript II reverse transcriptase, then incubated at 42°C for 60 min.

To synthesize double-stranded cDNA, the RNA-cDNA hybrid formed in the previous step was combined with 1× second strand buffer, 200 μM (ea.) dNTPs, 0.07 U/μL DNA ligase, 0.267 U/μL DNA polymerase I, and 0.0133 U/μL RNase H and incubated at 16°C for 2 hr. After 2 hr, the ends were polished with T4 DNA polymerase for 5 min. The reaction was stopped by adding 0.5 M EDTA. An equal volume of 25:24:1 phenol:chloroform:isoamyl alcohol (Fisher Scientific, Pittsburgh, PA, USA) was added and the solution was vortexed. The aqueous phase was removed and double-stranded cDNA was precipitated in ethanol.

The double-stranded cDNA was converted to cRNA by *in vitro *transcription using the Ambion MEGAscript T7 IVT kit (Austin, TX, USA). Double-stranded cDNA was combined with 7.5 mM ATP and GTP, 5.625 mM CTP and UTP, and 1.875 mM biotinylated UTP and biotinylated CTP (Perkin-Elmer, Wellesley, MA, USA). After mixing, the solution was incubated at 37°C for 5 hr. The cRNA was recovered using a RNeasy Mini Kit (Qiagen, Valencia, CA, USA). After elution of cRNA, the total volume of cRNA solution was 40 μL; 10 μL of 5× fragmentation buffer (Affymetrix, Santa Clara, CA, USA) was added and the solution was incubated at 95°C for 35 min. Fragmented cRNA was stored at -80°C until microarray hybridization.

### Microarray hybridization

This study used NimbleGen™ (Madison, WI, USA) maskless photolithographic, oligonucleotide microarrays [92]. The arrays were designed based on the complete *S. cerevisiae *genome sequence and annotation of 6,322 verified and hypothetical open reading frames (ORFs) obtained from the *Saccharomyces *Genome Database. A high-density oligonucleotide array was designed for this study containing 15 probe pairs per ORF, where each pair contains a perfect match (PM) oligonucleotide and a mismatch (MM) oligonucleotide. The PM sequence is a 24-mer oligonucleotide probe from the *S. cerevisiae *genome. MM probes were identical except for substitutions at positions 6 and 12 from the 5' end. To generate MM sequences, bases were systematically substituted by inversion as follows: A → T, C → G, G → C, and T → A. On each array, genes were represented by an identical number of oligos to minimize bias.

Four microarrays were hybridized at a time using the NimbleGen™ hybriwheel. Three arrays contained samples exposed to one of three doses of DNA-damaging agent (either MMS or γ-ray) and one was a control array with an untreated sample. Pre-hybridization solution consisting of 0.1 μg/μL herring sperm DNA, 0.5 μg/μL acetylated BSA, and 1× MES hybridization buffer was heated to 95°C for 5 min and then cooled to 45°C for 5 min. The solution was centrifuged at 12,000 g for 10 min. 400 μL of pre-hybridization solution was added to each array, followed by incubation at 45°C for 15 min. After the incubation, the pre-hybridization solution was removed from the arrays.

Hybridization solution consisting of 10 μg of cRNA, 0.33 pmole CPK6 oligos (NimbleGen™ Systems), 0.1 μg/μL herring sperm DNA, 0.5 μg/μL acetylated BSA, and 1× MES hybridization buffer was heated to 95°C for 5 min and then cooled to 45°C for 5 min. The solution was then centrifuged at 12,000 g for 10 min. 300 μL of hybridization solution was added to each microarray. The array was incubated for 16 hours at 45°C and 5 rpm on a hybriwheel.

After hybridization, the hybridization solution was removed and the arrays were rinsed 3× with 350 μL nonstringent wash buffer (6× SSPE, 0.01% Tween 20) (1× SSPE is 0.18 M NaCl, 10 mM NaH2PO4, and 1 mM EDTA [pH 7.7]) at room temperature. After the final rinse, arrays were incubated with 350 μL stringent wash buffer (0.1 M MES buffer [pH 6.5], 26 mM NaCl, 0.01% Tween 20) at 45°C for 30 min, during which the stringent wash buffer was replaced every 5 min. After the 30 min incubation, the stringent wash buffer was removed and 350 μL of 1× stain solution consisting of 1× stain buffer, 2 mg BSA, and Cy3 (Amersham, Piscataway, NJ, USA) was applied to each array. After the arrays were incubated with stain solution for 25 min at room temperature, they were rinsed with nonstringent wash buffer before being dipped in 1× NimbleGen™ final wash buffer for 30 seconds. Finally, the microarrays were blown dry with high pressure argon.

### Microarray data analysis

Hybridized microarrays were scanned using a Gene Pix 4000B scanner (Axon Instruments, Molecular Devices Corporation, Sunnyvale, CA, USA) and signal intensity values for each probe were extracted using NimbleScan software. For each probe pair, the MM signal was subtracted from the corresponding PM signal to reduce the effect of nonspecific hybridization signal when estimating transcript abundance. It has been noted that that this may be unnecessary [93] but results of a large number of 'spike-in' experiments suggest that subtraction of nonspecific signal is preferable and reduces false negative and false positive rates [94]. The data from the 15 probe pairs specific for each ORF were averaged, excluding any probe pairs where the signal was more than 2 standard deviations from the average of that probe set. For purposes of normalization of the array intensity data, array-specific scaling factors were calculated by assuming a constant mean signal intensity of 1,000 signal units for each array. The signal for each ORF was obtained by multiplying the raw signal intensity by the array-specific scaling factor. The ratio of signal for each ORF from an MMS or γ-ray treated sample was calculated relative to the signal from a paired untreated control sample. Ratios of signal intensities were log_2 _transformed for hierarchical clustering. All microarray data are available for public download through the Gene Expression Omnibus at  (Series GSE6018).

### Hierarchical clustering

Expression changes were estimated from three replicates of each experimental treatment for all 6,322 ORFs represented on the microarrays. False discovery rate (FDR) analysis as described by Storey [95] was used to filter gene responses for false positives. An FDR cutoff of 0.05 was applied, suggesting 1 false positive in every 20 genes considered to be differentially expressed. Storey's method is appropriate for comparing expression values on genome wide studies, since it allows one to control false positives without being so conservative that a large number of false negatives will be introduced. Genes that met this statistical significance criteria in at least one experimental treatment and changed by at least 3-fold in one experimental treatment were selected for hierarchical clustering (average-linkage clustering using uncentered Pearson correlation coefficients in Cluster [96]). All files generated from Cluster were viewed using Java Treeview.

Groups of co-regulated genes were identified by clustering patterns of changes in transcript levels across experimental conditions. Changes were measured for each gene as ratios of signal between experimental and control samples. This identified transcripts that show similar relative levels of change compared to the control, but does not capture differences in absolute RNA abundance levels between genes. An alternative clustering of genes based on the actual signal intensity values, either Perfect Match minus Mismatch or Perfect Match only, could be used to identify genes with similar expression levels across conditions.

### MEME system motif analysis

The MEME system was used to search for highly conserved regions of DNA upstream from clusters of coregulated genes [55]. DNA sequences containing the 500 bp upstream from each gene represented in a cluster were simultaneously analyzed with the MEME parameters of minimum width of 5 bp, maximum width of 20 bp, and maximum number of motifs to find of 6. Only enriched regions with E-values of 0.10 or less are reported in the text.

The position-specific probability matrices of conserved motifs identified with MEME were compared to known motifs in the JASPAR database [97]. This comparison used a modified Needleman-Wunsch algorithm as previously described [98]. The comparison yielded a raw score (where the maximum score is twice the width of the shorter motif) and a percentage of maximum score achieved. The highest score for human and rat or mouse genes are reported in the text.

## Authors' contributions

MGB performed the hybridizations and hierarchical clustering. SS helped conceive methods for the hybridization and data analysis. JDG contributed to the design of the microarrays and analysis of the data. SPP conceived the study and directed its execution. All authors read and approved the final manuscript.

## Supplementary Material

Additional file 1 **Log_2 _fold change data for all arrays**. This text file contains the log_2 _fold change value for all 6,322 ORFs represented on the NimbleGen™ arrays. There are 18 columns of data, 3 for each MMS dose (0.001%, 0.01%, and 0.1%) and 3 for each γ-ray dose (1 Gy, 10 Gy, 100 Gy). Click here for file

Additional file 2 **Average log_2 _fold change for all genes with significant transcriptional response to MMS or γ-ray**. This EXCEL file contains the log_2 _fold change value for all 920 genes with a statistically significant transcriptional response to MMS and/or γ-ray. There are 6 columns of data, 3 with the average log_2 _fold change for each dose of MMS and 3 with the average log_2 _fold change for each dose of γ-ray. These genes were clustered and shown in Figure 1. Additionally, this file contains the GO annotation assigned to each gene and shown in Figure 7-A. Click here for file

Additional file 3 **Average log_2 _fold change for all genes with a significant transcriptional response to MMS**. This EXCEL file contains the log_2 _fold change value for all 714 genes with at least a 3-fold transcriptional response to MMS. There are 3 columns of data, each one containing the average log_2 _fold change for one dose of MMS. These genes were clustered and shown in Figure 5-A. Additionally, this file contains the GO annotation assigned to each gene and shown in Figure 7-B. Click here for file

Additional file 4 **Average log_2 _fold change for all genes with a significant transcriptional response to γ-ray**. This EXCEL file contains the log_2 _fold change value for all 482 genes with at least a 3-fold transcriptional response to γ-ray. There are 3 columns of data, each one containing the average log_2 _fold change for one dose of γ-ray. These genes were clustered and shown in Figure 5-B. Additionally, this file contains the GO annotation assigned to each gene and shown in Figure 7-C. Click here for file

Additional file 5 **MEME system motif analysis results**. This EXCEL file contains the 3 enriched motifs identified in the text from Figure 3-D, Figure 4, and Figure 6-B. The columns contain the motif and its E-value as well as the ORF, gene product, and GO annotation for each gene associated with the enriched region. Click here for file

Additional file 6 **GO analysis of clusters presented in **Figure 5. This figure contains the results of gene ontology analysis of the clusters presented in Figure 5. (A) genes induced by MMS, (B) genes repressed by MMS, (C) genes induced by γ-ray, and (D) genes repressed by γ-ray. Genes are sorted according to their GO biological process annotation and can be viewed in Additional Files 3 (MMS) and 4 (γ-ray). Click here for file

Additional file 7 **Cell viability after exposure to DNA-damaging agent**. This table shows the percentage of *Saccharomyces cerevisiae *cells living one hour after exposure to the DNA damage caused by MMS or γ-ray. Viability was determined by flow cytometry quantifying the percentage of cells excluding propidium iodide. Click here for file
