# Supplementary figures and images for: Analyzing the dose-dependence of the Saccharomyces cerevisiae global transcriptional response to methyl methanesulfonate and ionizing radiation
Source: BMC Genomics. 2006 Dec 1;7:305. doi: 10.1186/1471-2164-7-305 (PMC1698923; doi:10.1186/1471-2164-7-305)

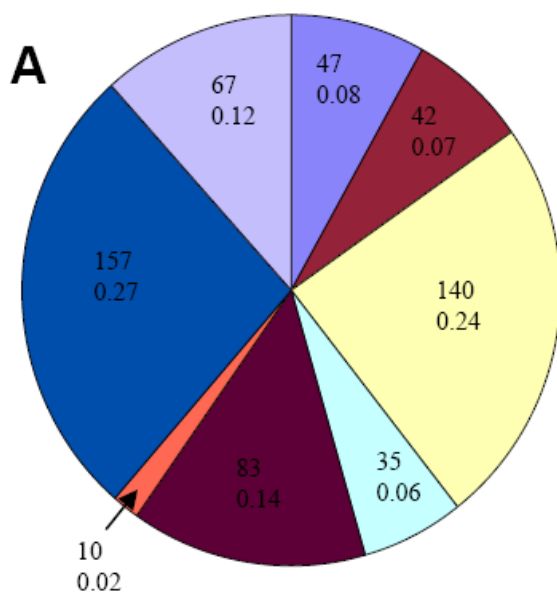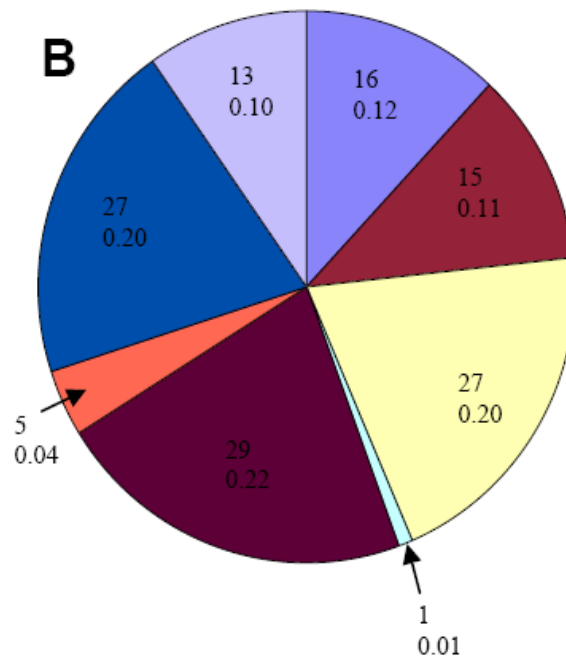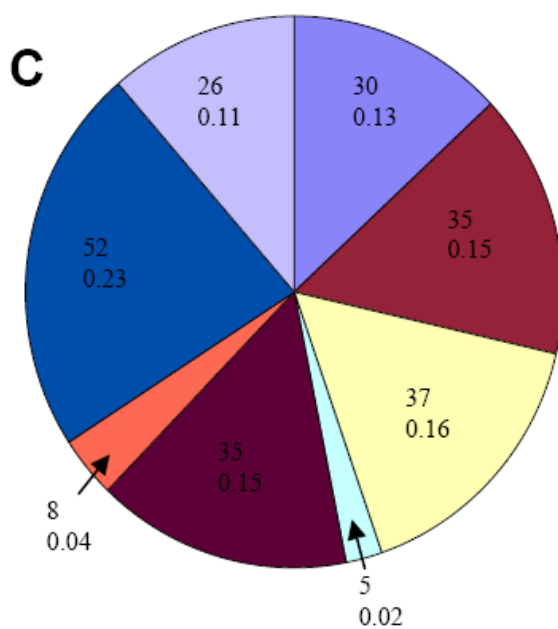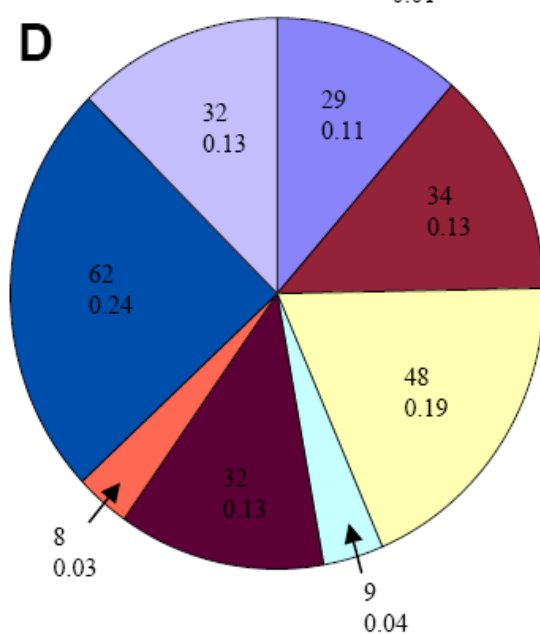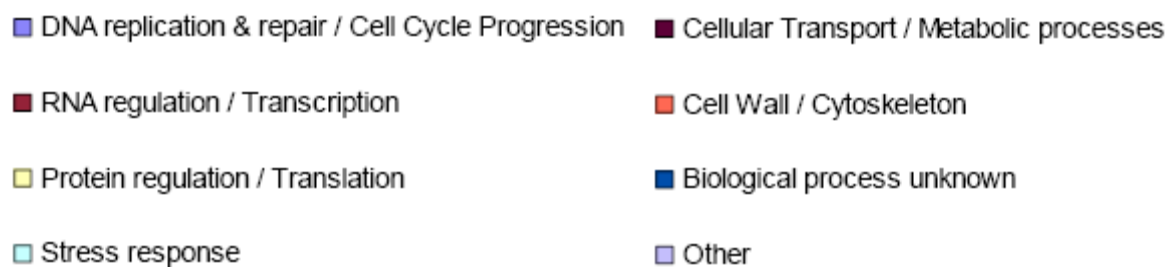

Supplement: Additional file 6 — GO analysis of clusters presented in Figure 5. This figure contains the results of gene ontology analysis of the clusters presented in Figure 5. (A) genes induced by MMS, (B) genes repressed by MMS, (C) genes induced by γ-ray, and (D) genes repressed by γ-ray. Genes are sorted according to their GO biological process annotation and can be viewed in Additional Files 3 (MMS) and 4 (γ-ray). [file 1471-2164-7-305-S6.pdf]
