# Supplementary material for: Analyzing the dose-dependence of the Saccharomyces cerevisiae global transcriptional response to methyl methanesulfonate and ionizing radiation
Source: BMC Genomics. 2006 Dec 1;7:305. doi: 10.1186/1471-2164-7-305 (PMC1698923; doi:10.1186/1471-2164-7-305)
Supplement: Additional file 7 — Cell viability after exposure to DNA-damaging agent. This table shows the percentage of Saccharomyces cerevisiae cells living one hour after exposure to the DNA damage caused by MMS or γ-ray. Viability was determined by flow cytometry quantifying the percentage of cells excluding propidium iodide. [file 1471-2164-7-305-S7.pdf]

Supplemental Table – Cell viability after exposure to DNA-damaging agent

Damaging Agent	Dose	Percent Living Cells
MMS	0%	98.9 ± 1.1
	0.001%	98.2 ± 1.2
	0.01%	99.1 ± 0.6
	0.1%	98.7 ± 0.6
γ-ray	0 Gy	99.5 ± 0.1
	1 Gy	99.6 ± 0.2
	10 Gy	99.2 ± 0.7
	100 Gy	99.1 ± 0.5

Percentage of *Saccharomyces cerevisiae* cells living one hour after exposure to the DNA-damaging agent listed. Viability was determined by flow cytometry quantifying the percentage of cells excluding propidium iodide. The average of three independent experiments is shown, with the standard deviation for each dose.
